# The PRESEP score: an early warning scoring system to identify septic patients in the emergency care setting

**DOI:** 10.1186/cc14022

**Published:** 2014-12-03

**Authors:** O Bayer, CS Hartog, D Schwarzkopf, C Stumme, A Stacke, F Bloos, C Hohenstein, B Kabisch, C Weinmann, J Winning, Y Sakr, JK Reinhart

**Affiliations:** 1 Department of Anaesthesiology and Intensive Care Medicine, Jena University Hospital, Jena, Germany; 2 Center for Sepsis Control & Care, Jena University Hospital, Jena, Germany; 3 Emergency Department, Jena University Hospital, Jena, Germany

## Introduction

Many patients present with sepsis through emergency services [1]. Their outcome could be improved if sepsis could be detected already in the prehospital setting. This study aims to develop and evaluate a score to detect prehospital early sepsis.

## Methods

A retrospective study of 375 patients admitted to Jena University Hospital emergency department (ED) through emergency medical services (EMS). Sepsis was present in the ED in 93 (24.8%) patients, of which 60 (16.0%) had severe sepsis and 12 (3.2%) had septic shock. The following predictors for sepsis based on consensus criteria were extracted from the EMS protocol: body temperature (T), heart rate (HR), respiratory rate (RR), oxygen saturation (SaO_2_), Glasgow Coma Scale, blood glucose and systolic blood pressure (BP). Sepsis predictors were determined based on inspection of loess graphs. Backward model selection was performed to select risk factors for the final model. The PRESEP score was calculated as the sum of simplified regression weights. Its predictive validity was compared to the modified Early Warning Score (MEWS) [2], the Robson screening tool [3] and the BAS 90-30-90 [4].

## Results

Backward model selection identified T, HR, RR, SaO_2 _and BP for inclusion in the PRESEP score. Its AUC was 0.93 (CI 0.89 to 0.96). The cutoff based on maximum Youden's Index was ≥4 (sensitivity 0.85, specificity 0.86, PPV 0.66, NPV 0.95). The PRESEP score had a larger AUC than the MEWS (0.93 vs. 0.77, *P *< 0.001) and surpassed MEWS and BAS 90-60-90 concerning sensitivity (0.74, 0.62), specificity (0.75, 0.83), PPV (0.45, 0.51) and NPV (0.91, 0.89). The Robson screening tool had a higher sensitivity and NPV (0.95, 0.97) was better, but its specificity and PPV lower (0.43, 0.43).

## Conclusion

The PRESEP score can be easily applied in the emergency setting and could be a valuable tool to identify septic patients in the case of suspected infection.
